# Understanding the Time Needed to Link to Care and Start ART in Seven HPTN 071 (PopART) Study Communities in Zambia and South Africa

**DOI:** 10.1007/s10461-018-2335-7

**Published:** 2018-11-10

**Authors:** Janet Seeley, Virginia Bond, Blia Yang, Sian Floyd, David MacLeod, Lario Viljoen, Mwelwa Phiri, Melvin Simuyaba, Graeme Hoddinott, Kwame Shanaube, Chiti Bwalya, Laing de Villiers, Karen Jennings, Margaret Mwanza, Ab Schaap, Rory Dunbar, Kalpana Sabapathy, Helen Ayles, Peter Bock, Richard Hayes, Sarah Fidler, the HPTN 071 (PopART) study team

**Affiliations:** 1 London School of Hygiene and Tropical Medicine, London, UK; 2 Zambart, School of Medicine, University of Zambia, Ridgeway Campus, Lusaka, Zambia; 3 Department of Paediatrics and Child Health, Faculty of Medicine and Health Sciences, Desmond Tutu TB Centre, Stellenbosch University, K-Floor, Clinical Building, Tygerberg Medical Campus, Francie van Zyl Drive, Tygerberg, 7505 South Africa; 4 City of Cape Town Health Directorate, Cape Town, South Africa; 5 Zambia Prevention Care and Treatment partnership (ZPCT), Lusaka, Zambia; 6 Department of Medicine, Imperial College, London, UK

**Keywords:** HIV, Anti-retroviral treatment, Access, Linkage to care and ART, Southern Africa

## Abstract

To achieve UNAIDS 90:90:90 targets at population-level, knowledge of HIV status must be followed by timely linkage to care, initiation and maintenance of antiretroviral therapy (ART) for all people living with HIV (PLHIV). Interpreting quantitative patterns using qualitative data, we investigate time taken to link to care and initiate ART amongst individuals aware of their HIV-status in high HIV-prevalence urban communities in the HPTN 071 (PopART) study, a community-randomised trial of a combination HIV prevention package, including universal testing and treatment, in 21 communities in Zambia and South Africa. Data are drawn from the seven intervention communities where immediate ART irrespective if CD4 count was offered from the trial-start in 2014. Median time from HIV-diagnosis to ART initiation reduced after 2 years of delivering the intervention from 10 to 6 months in both countries but varied by gender and community of residence. Social and health system realities impact decisions made by PLHIV about ART initiation.

**Electronic supplementary material:**

The online version of this article (10.1007/s10461-018-2335-7) contains supplementary material, which is available to authorized users.

## Background

The dual role of anti-retroviral treatment (ART) for HIV treatment and prevention is well established [1–3]. In 2014 UNAIDS and partners set global HIV testing and ART coverage targets to drive policy makers towards enhancing access to treatment for all people living with HIV (PLHIV) [4]. This change followed the findings of two randomised trials, START [1] and TEMPRANO ANRS [5] demonstrating enhanced survival with immediate versus deferred ART. The individual and public health benefits of these findings are embedded in the 2015 World Health Organization and, subsequently, national ART guidelines [3, 6–9]. As a result, messaging around the role of ART for PLHIV has changed dramatically in the past 3 years, from ART only for those with CD4 counts below a certain threshold to a recommendation of treatment, for all PLHIV irrespective of CD4 count [6, 9, 10].

Although mathematical models predicted that a public health approach of universal HIV testing with immediate ART treatment for all identified HIV-positive individuals has the capacity to reduce HIV incidence [11], the population-wide delivery of this strategy is challenging [12]. This is particularly so in high burden, resource-constrained settings [13–15] despite some recent encouraging results from combination interventions [16, 17].

In this paper, we report findings on time taken to link to care from the HPTN 071 (PopART) study. This is a community randomised trial in 21 large, urban and peri-urban, resource-constrained communities with high HIV prevalence (20%) in Zambia and South Africa [18] with a total community population of one million people. The trial is evaluating the effect of a combination HIV prevention package including the Universal Test and Treat (UTT) strategy on population level HIV incidence. The intervention was delivered to each household within a community by a cadre of trained lay-counsellors (Community HIV care Providers [CHiPs]). The CHiPs attempted to visit every household within a community on an annual basis (called a “Round”), for three complete Rounds, offering HIV education, HIV testing and linkage to care and treatment for HIV-positive individuals. In seven intervention (arm A) communities, from December 2013 (prior to the implementation of changes in WHO and national ART guidelines in 2016) to December 2017, the PopART HIV prevention combination package included the offer of ART regardless of CD4 count to all PLHIV who knew their HIV-positive status.

For PLHIV, delays to treatment uptake have been documented in many different settings, including clinical trials. The HPTN 052 trial, undertaken at a time when ART initiation guidelines remained linked to a CD4 threshold, was designed to determine the effect of ART on the transmission of HIV from PLHIV to their HIV-negative sexual partners. The trial began in 2005, and was stopped early for efficacy, following an interim analysis in 2011. Of relevance to the focus of this paper, following the decision to stop the protocol early, all HIV-positive study participants randomised to the trial deferred treatment arm were offered ART, regardless of their CD4 count. One year later, despite counselling of participants and provision of access to ART, 17% had not taken up treatment and by 2015, on completion of the trial, 4% remained off treatment [2]. This observation identified that although the participating PLHIV were well-informed and supported, some still needed and took time to start treatment, reasoning that they were too healthy to start ART, their CD4 count was not low enough, and/or they feared drug side effects [2]. This evidence that some PLHIV needed time to start ART has been mirrored in several other studies [19–27]. The ANRS 12249 TasP trial [15] in KwaZulu-Natal (South Africa), despite high uptake of community HIV testing, had challenges with linkage to care and ART initiation, showing that only 36.9% linked to care by 3 months after referral [28]. Some factors resulting in delayed uptake of ART are linked to broader health service delivery challenges that impede progress in providing treatment to everyone living with HIV, including access to and availability of drugs [29–33]. The barriers to treatment associated with the health service may be compounded by, for example, the persistence of HIV-related stigma which affects a person’s ability to access care and other demands on time due to making a livelihood or caring for children at home [34, 35].

We have previously reported the coverage of HIV testing [36] and ART [37], and estimates of the time from CHiP referral to linkage to HIV care and ART initiation during the first annual Round in the four Zambian arm A (intervention) communities. Although we showed significant improvements in knowledge of HIV status and ART coverage by the end of Round 1 (the first year) of intervention, the median time from CHiP referral to ART initiation was much longer than had been anticipated, at ~ 10 months [37]. Subsequently, towards the end of that annual Round 1 concerted efforts were made to facilitate more rapid linkage to care.

Following these efforts, we were able to estimate the time taken from CHiP referral to ART initiation during the second annual Round (the second year) of the intervention (June 2015–October 2016), across the four Arm A communities in Zambia and in addition the 3 Arm A communities in South Africa. In this paper we draw on these longitudinal quantitative process data from Round 2, together with trial qualitative data, to address the question of why some PLHIV are less able or likely to start ART quickly even after 1 year’s experience of delivering UTT, and in the context of support for linkage to care from CHiPs. Specifically, we describe intervention efforts to reduce the lag in linkage to care, present quantitative data on time to linkage to care and time to ART initiation, and through qualitative data provide the explanations given by PLHIV for the choices they have made about whether and when to link to HIV care and initiate ART. Finally, we use the mixed methods evidence to reflect on current policy directions, particularly the strong global emphasis on commencing ART on the same day as first testing HIV-positive.

## Methods

### Setting

For the HPTN 071 (PopART) trial intervention, a community was defined as the population catchment of a government health care facility providing HIV services including ART. The seven communities included in this analysis were randomly allocated to receive the full PopART intervention package, including offer of ART initiation regardless of CD4 count for PLHIV from the start of the trial, and prior to changes in guidelines. The communities included have a total population of ~ 200,000 in the four Zambian communities, and ~ 100,000 in the three South African communities. The social context and HIV services in all the communities in both countries have been described elsewhere [38]. An ancillary study on stigma was undertaken, which provides additional information on stigma experiences in health facilities and levels of stigma in different communities, also described elsewhere [35, 39, 40].

The PopART intervention was a combination HIV prevention package delivered by community HIV care providers (CHiPs) and is described in detail in Hayes et al. [18], and summarised here. CHiPs visited every household within a community annually offering HIV education and testing using routine point of care finger prick rapid testing [41] to all consenting household members. ART was provided by local government health care facilities. For all individuals newly-testing HIV-positive or reporting that they know they are HIV-positive but not yet on ART, the CHiPs through repeated household visits, encouraged and facilitated linkage to care for ART initiation at the local clinic. The ART drugs were provided through the routine health care system funded by PEPFAR-supported implementing partners. It should be noted that until the change in national guidelines in 2016 (recommending prompt treatment, regardless of CD4 count), Arm A participants had to sign a consent form if their CD4 count was > 500, prior to initiation of ART. Because they had to wait for their CD4 count results, there was a brief delay of approximately 2 weeks between linkage to care and ART start.

In Round 2, several new strategies were put in place to encourage linkage to care, to be undertaken by the CHiPs, and existing practices from Round 1 were reinforced [42]. These included assisted referrals: CHiPs escorting clients to the clinic to help them link to care and specially trained counsellors in each community to assist with linkage; working with existing community health care workers to track clients; following up with clients who missed clinic appointments and holding meetings with local clinic staff on a regular standardised basis to review which CHiP clients had, or had not, linked to care and/or started ART.

### Quantitative Data

In order to offer annual HIV testing as well as other HIV prevention services, CHiPs aimed to contact all individuals (aged ≥ 15 years) at least once every round to offer participation in the intervention. All household members were asked for verbal informed consent to take part in the intervention and permission to collect data electronically. Individuals provided written consent for HIV counselling and testing. CHiPs recorded basic data on the household and more detailed information for individuals who consented to participate in the intervention. Data captured electronically included age and gender, self-reported HIV status, the outcome of HIV testing by CHiPs, self-reported information on HIV care registration and current (during the last 1 month) ART use, and whether a referral to HIV care was given. Analysis was restricted to participants aged 15 years and older, although HIV testing was offered irrespective of age.

Individuals were “known HIV-positive” to the CHiPs in Round 2 if they participated in Round 2 and either (i) had self-reported or tested HIV-positive in Round 1, and verbally confirmed their HIV-positive status in Round 2 (ii) self-reported HIV-positive for the first time in Round 2, or (iii) tested HIV-positive with CHIPs in Round 2. For individuals who self-reported they had previously registered for HIV care, CHIPs asked to see the patient ART card, and if the card was provided then the ART card number was recorded.

For all individuals known to be HIV-positive by the definition above, CHIPs undertook follow-up visits to support linkage to care and subsequent ART initiation, as well as to collect self-reported information on whether and when these outcomes were achieved. If the individual was not at home at the time of the CHiP follow-up visit, then the CHiP recorded that they had made a visit but were not able to meet the individual.

Analyses of the time from CHiP referral to linkage to care, from CHIP referral to ART initiation, and the time from linkage to care to ART initiation, were done using the Kaplan–Meier “time-to-event” method. We used Cox regression for analysis of whether these times differed by country, community, gender or age group. We used follow-up data to 30th September 2017 and for individuals who were not known to have started ART by this date, we censored their follow-up on the last date on which they met the CHiP in person. Individuals do not contribute to the analysis if they were never followed up in person after they were referred to care.

### Qualitative Data

Social science enquiry encompassed qualitative research prior to the intervention period (2012–13) and during the intervention. The trial qualitative research used ethnographic and participatory research methods [43] and was designed to first document rapidly key features of study communities of relevance to HIV prior to the intervention period through broad brush surveys (BBS) [44], and then, during the intervention, focus on community responses, implementation and different trajectories around a range of HIV outcomes and decisions (labelled ‘qualitative cohort’). In this paper, we draw on findings on community characteristics from the BBS (the methods are described in detail in other papers, see [43, 44]) and on findings from a sub-sample of PLHIV in the qualitative cohort in both countries documenting the challenges in linkage to care alongside other experiences. The research was carried out by a core team of Zambian and South African men and women social scientists trained in qualitative data collection methods, who were competent in the appropriate local languages. In both countries, individual social scientists were allocated to three to four communities where they, and a field research assistant, conducted all research components as a pair, building their familiarity with particular communities and families over time. Efforts were made to have each pair composed of a man and a woman where possible.

The aim of the qualitative cohort research was to recruit participants purposively sampled to represent different HIV-related decisions and outcomes across different trial arms, geographical locations, age, gender, and key populations. Sixty-eight households (21 in Zambia and 47 in South Africa) where PLHIV resided were recruited from March 2016 to November 2017 (a time period which overlapped with the quantitative data collection in Rounds 2 and 3). Two-thirds of the PLHIV in the households included in the cohort were on ART. Of the other third, half had defaulted from treatment and half were yet to start ART. Participants in the cohort were interviewed at least three times, and households visited at least six times over a year. The interviewers asked about family, kin and social networks, movement within and outside of the household and community, household livelihood, HIV and general health service access, sex, love and romance, and future ambitions and fears. Interviews were recorded, and notes were also taken during and after interviews, with each social scientist first writing up a reflective summary of the findings and then debriefing with other team members, before translating and transcribing the interviews. ATLAS-ti qualitative analysis software was used to manage the data.

This body of data was closely reviewed and analysed by the social science team for this paper, focusing on the participants who were living with HIV from across all seven Arm A communities with the aim of explaining time between HIV diagnosis and ART initiation. Notes from field work, summaries, debriefing sessions and transcriptions were scrutinised. This was done by first holding two-day analysis workshops in both countries with the social scientists who carried out the fieldwork. This process allowed key themes to emerge across both countries (for example, `gender patterns and differences’, `when it makes sense to start ART on the same day’), which were then shared to arrive at a shared set of codes. The teams were asked to review the data to code against the themes, ensuring that coding was cross checked for consistency across coders throughout the process. Each country produced a synopsis document, which was discussed by the country teams highlighting differences and similarities, to arrive at the list of themes (shown in Tables 3 and 4) used in this paper.

### Ethical Considerations

The HPTN 071 (PopART) study was approved by the London School of Hygiene and Tropical Medicine, University of Zambia, and Stellenbosch University (N12/11/074), ethics committees and by other health governmental authorities. Research participants signed written informed consent. Those who consented to a finger prick rapid test signed written consent according to standard government guidelines. Data from the CHiP intervention were collected following verbal consent.

To ensure confidentially, quotations from the qualitative data are only labelled with Z = Zambia and SA = South Africa, and community number.

## Results

### Quantitative Findings

**Fig. 1:**
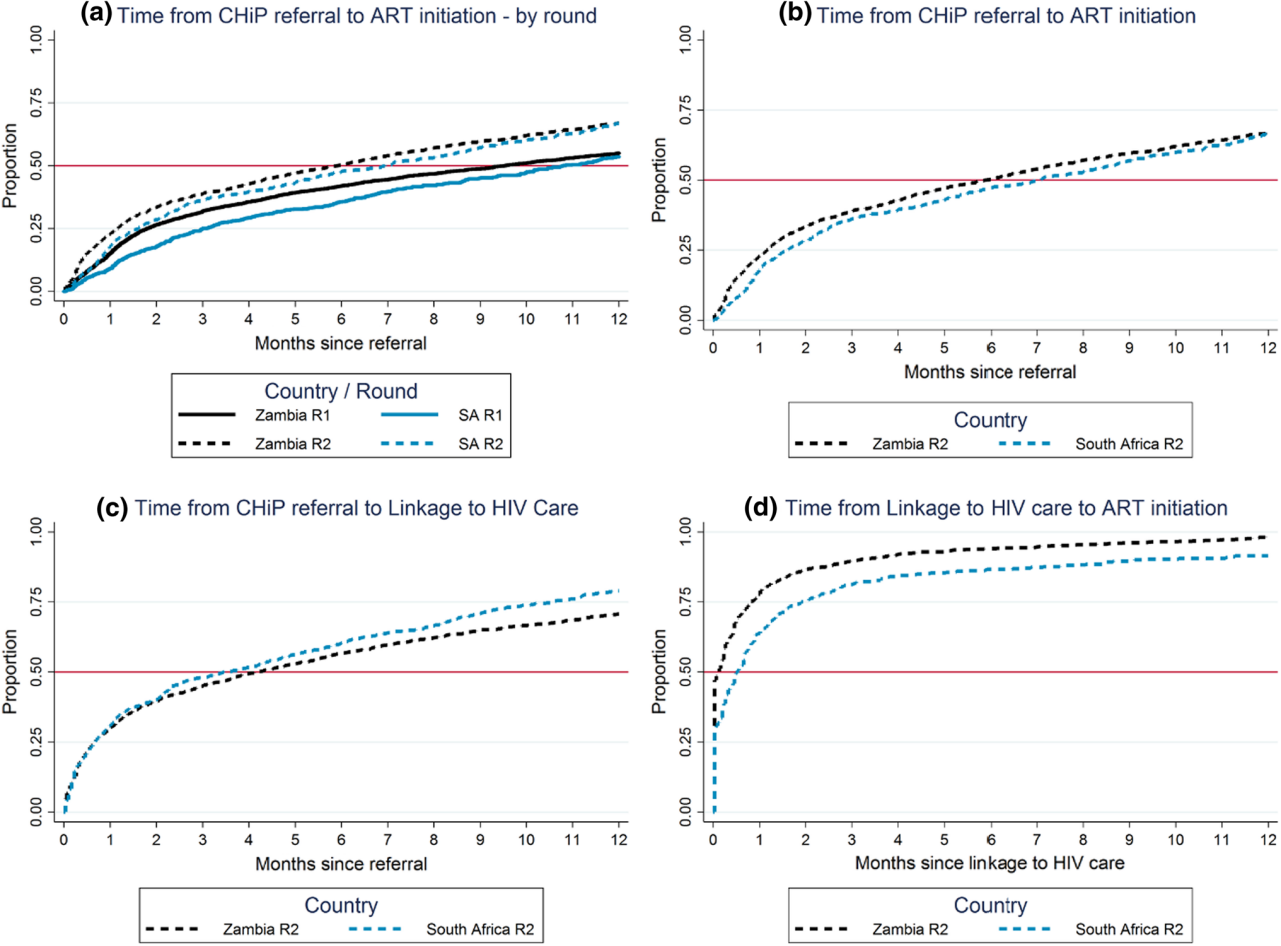
Time from CHiP referral to ART initiation, time from CHiP referral to linkage to HIV care, and time from linkage to HIV care to ART initiation

During Round 2, in Zambia 3435 and in South Africa 1262 HIV-positive individuals were referred to HIV care by CHiPs, among those who were not on ART on the date of first participation in Round 2. Overall, the estimated median time from CHIP referral to ART initiation was ~ 6 months in Round 2 in both Zambia and South Africa, considerably less than the median of ~ 10 months in both countries in Round 1 but still slower than had been targeted at the start of the intervention (Fig. 1a; the horizontal red line is drawn at 50% to correspond to the median time to ART initiation).

### Time from CHiP Referral to ART Initiation—Overall Estimates

Overall, we estimated that 39, 50, and 67% initiated ART by 3, 6, and 12 months respectively after first referral to care by CHiPs in Round 2 in Zambia (Fig. 1b), with corresponding (and slightly lower) estimates of 36, 47, and 66% in South Africa (Fig. 1b).

### Time from CHiP Referral to Linkage to Care (Attendance at Clinic)—Overall Estimates

The estimates for the percentage who were linked to care, i.e. first attended a clinic, by 3, 6 and 12 months was 45, 57 and 71% respectively after first referral to care in Zambia, and 48, 60, and 79% in South Africa (Fig. 1c); these estimates indicated slightly more rapid linkage to care in South Africa compared with Zambia.

### Time from Linkage to Care (Attendance at Clinic) to ART Initiation

Notwithstanding the imprecision around our estimates of both the date of linkage to care and the date of ART initiation, because both dates were based on self-reported information, we also estimated the time from linkage to care to ART initiation among individuals who linked to care. We estimated that 78, 90, and 94% started ART by 1, 3, and 6 months respectively after linkage to care in Zambia, with corresponding (and slightly lower) estimates of 64, 81, and 87% in South Africa (Fig. 1d).

### Time from Linkage to Care (Attendance at Clinic) to ART Initiation, Relative to Time from CHiP Referral to Linkage to Care

Overall, for most individuals the time from linkage to care to ART initiation was short, with a median of less than 1 month (Fig. 1d), while the time from referral to linkage to care was longer, with a median of 4 months in Zambia and 3.5 months in South Africa (Fig. 1c).

### Time from CHiP Referral to ART Initiation—Patterns by Gender, Community, and Age Group

Patterns by gender, community and age group for the time from CHiP referral to ART initiation were similar to those for the time from CHiP referral to linkage to care (attendance at clinic). Here we have summarized the patterns for our estimates of time to ART initiation (Tables 1 and 2), with estimates for time from CHiP referral to linkage to care provided in Supplementary Table 1.

**Table 1:** Time to initiate ART after first CHiP referral to HIV care in Round 2—Zambia

	Number referred to HIV care	ART initiated (%)^a^				Hazard ratio, unadjusted	Hazard ratio, adjusted^b^	95% CI
		1 month	3 months	6 months	12 months			
Overall	3435	23 95% CI 21–24 (*n *= 2570)^c^	39 95% CI 37–41 (*n *= 2347)	**50** 95% CI 48–52 (*n *= 2188)	**67** 95% CI 65–69 (*n *= 1973)			
Gender								
Men	1117	26	44	**56**	**71**	1 (ref)	**1 (ref)**	*p *< 0.001^d^
Women	2318	21	37	**48**	**65**	0.82	**0.82**	**0.73**–**0.91**
Community								
1	256	39	54	**65**	**78**	1.52	**1.55**	**1.29**–**1.87**
2	856	21	39	**51**	**67**	0.95	**0.94**	**0.83**–**1.07**
3	1775	24	40	**52**	**69**	1 (ref)	**1 (ref)**	*p *< 0.001
4	548	14	26	**38**	**56**	0.67	**0.68**	**0.58**–**0.79**
Men, age group (years)								
15–19	29	33	39	**63**	**69**	1.01	**0.95**	**0.54**–**1.67**
20–24	100	17	31	**46**	**59**	0.70	**0.72**	**0.50**–**1.03**
25–34	453	29	46	**54**	**67**	1 (ref)	**1 (ref)**	*p *= 0.04
35–44	369	25	43	**57**	**74**	1.03	**1.06**	**0.86**–**1.31**
45–54	132	25	47	**58**	**79**	1.17	**1.18**	**0.90**–**1.55**
55 +	34	26	71	**79**	**/** ^e^	1.71	**1.75**	**1.11**–**2.75**
Women, age group (years)								
15–19	152	23	40	**53**	**74**	1.17	**1.10**	**0.84**–**1.43**
20–24	556	19	37	**48**	**67**	1.03	**1.03**	**0.88**–**1.21**
25–34	968	21	36	**47**	**65**	1 (ref)	**1 (ref)**	*p *= 0.24
35–44	428	22	37	**51**	**66**	1.03	**1.04**	**0.88**–**1.23**
45–54	154	26	37	**45**	**58**	0.90	**0.89**	**0.70**–**1.15**
55 +	60	19	29	**35**	**51**	0.67	**0.66**	**0.44**–**0.99**

Hazard ratios and their 95% CI, and estimates of the percentage of individuals with the outcome by key time points, are shown in bold

^a^Estimated from “time to event” analysis

^b^For overall comparison of women with men, adjusted hazard ratios are obtained from a multivariable Cox regression model including community, age group, and gender; for overall comparison among communities, adjusted hazard ratios are obtained from a multivariable Cox regression model including community, gender, and gender-specific hazard ratios for age group; for comparison across age groups, adjusted hazard ratios are obtained from gender-specific multivariable Cox regression models including community and age group; age-specific estimates are presented separately for men and women because there was statistical evidence the age pattern was different for men and women (p = 0.004)

^c^Number who either started ART within 1 month after referral or have a follow-up visit ≥ 1 month after referral, and similarly for other time points (3, 6, 12 months after referral)

^d^p-values are from Cox regression, from likelihood ratio tests of whether there is evidence of association between an individual characteristic (e.g. gender, or the community in which an individual lives) and the outcome of “time to ART initiation”

^e^/ = Cannot be estimated, because no one followed up to this time point

**Table 2:** Time to initiate ART after first CHiP referral to HIV care in Round 2—South Africa

	Number referred to HIV care	ART initiated (%)^a^				Hazard ratio, unadjusted	Hazard ratio, adjusted^b^	95% CI
		1 month	3 months	6 months	12 months			
Overall	1262	18 95% CI 15–20 (*n *= 855)^c^	36 95% CI 33–40 (*n *= 788)	**47** 95% CI 44–51 (*n *= 723)	**66** 95% CI 63–70 (*n *= 644)			
Gender								
Men	436	16	31	**42**	**58**	1 (ref)	**1 (ref)**	*p *= 0.006^d^
Women	826	19	39	**50**	**71**	1.33	**1.32**	**1.08**–**1.61**
Community								
Community 1	204	25	37	**53**	**72**	1.30	**1.23**	**0.96**–**1.57**
Community 2	909	14	34	**45**	**63**	1 (ref)	**1 (ref)**	*p *= 0.007
Community 3	149	31	49	**56**	**76**	1.54	**1.48**	**1.15**–**1.90**
Men, age group (years)								
15–19	5	33	67	**/** ^e^	**/**	4.70	**4.85**	**1.51**–**15.61**
20–24	44	18	26	**40**	**47**	0.91	**0.88**	**0.46**–**1.67**
25–34	195	14	25	**35**	**56**	1 (ref)	**1 (ref)**	*p *= 0.04
35–44	135	12	33	**43**	**57**	1.10	**1.02**	**0.69**–**1.52**
45–54	48	30	44	**62**	**70**	1.76	**1.60**	**1.00**–**2.57**
55 +	9	14	57	**57**	**/**	3.01	**2.51**	**1.07**–**5.92**
Women, age group (years)								
15–19	48	6	28	**36**	**48**	0.59	**0.58**	**0.32**–**1.05**
20–24	188	19	35	**45**	**75**	0.97	**0.98**	**0.75**–**1.29**
25–34	369	17	41	**51**	**70**	1 (ref)	**1 (ref)**	*p *= 0.42
35–44	149	24	42	**54**	**70**	1.03	**1.01**	**0.75**–**1.36**
45–54	54	23	37	**54**	**76**	1.08	**1.03**	**0.70**–**1.53**
55 +	18	25	57	**71**	**71**	1.40	**1.41**	**0.66**–**3.01**

Hazard ratios and their 95% CI, and estimates of the percentage of individuals with the outcome by key time points, are shown in bold

^a^Estimated from “time to event” analysis

^b^For overall comparison of women with men, adjusted hazard ratios are obtained from a multivariable Cox regression model including community, age group, and gender; for overall comparison among communities, adjusted hazard ratios are obtained from a multivariable Cox regression model including community, gender, and gender-specific hazard ratios for age group; for comparison across age groups, adjusted hazard ratios are obtained from gender-specific multivariable Cox regression models including community and age group; age-specific estimates are presented separately for men and women because there was weak statistical evidence the age pattern was different for men and women (p = 0.06)

^c^Number who either started ART within 1 month after referral or have a follow-up visit ≥ 1 month after referral, and similarly for other time points (3, 6, 12 months after referral)

^d^p-values are from Cox regression, from likelihood ratio tests of whether there is evidence of association between an individual characteristic (e.g. gender, or the community in which an individual lives) and the outcome of “time to ART initiation”

^e^/ = Cannot be estimated, because no one followed up to this time point

**Fig. 2:**
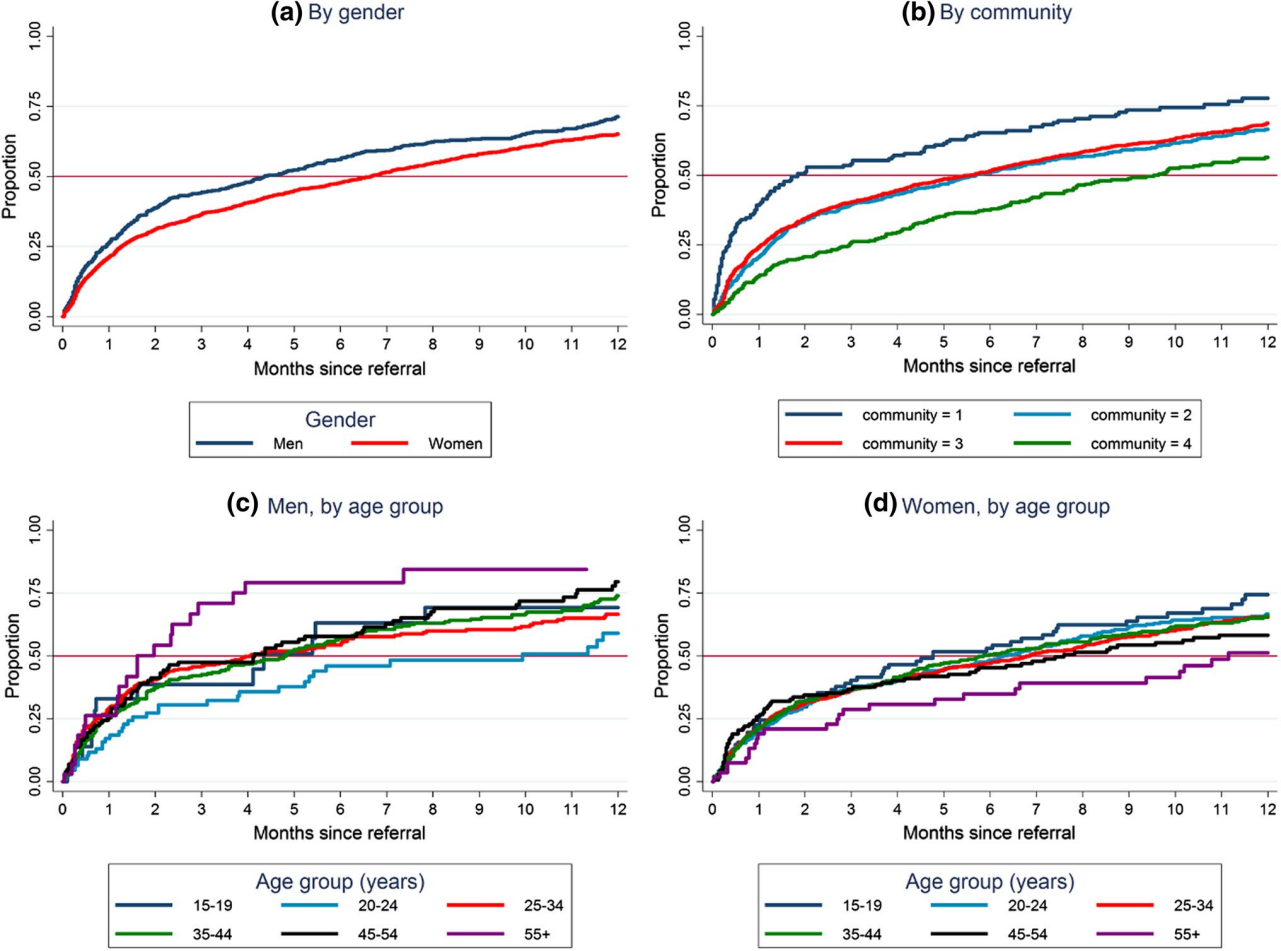
Time from CHiP referral to ART initiation in Zambia in Round 2, by gender, community, and age group

For Zambia, findings are summarised in Table 1 and Fig. 2. The median time to ART initiation was longer for women than men (6.6 and 4.4 months respectively, p < 0.001). There was strong evidence of differences between communities (p < 0.001), with the median time to ART initiation being similar in communities 2 and 3 (5.7 and 5.6 months respectively) but shorter in community 1 (1.8 months) and longer in community 4 (9.5 months). Among men, it was longest for those aged 20–24 years, and among women it was longest for those aged ≥ 55 years.

**Fig. 3:**
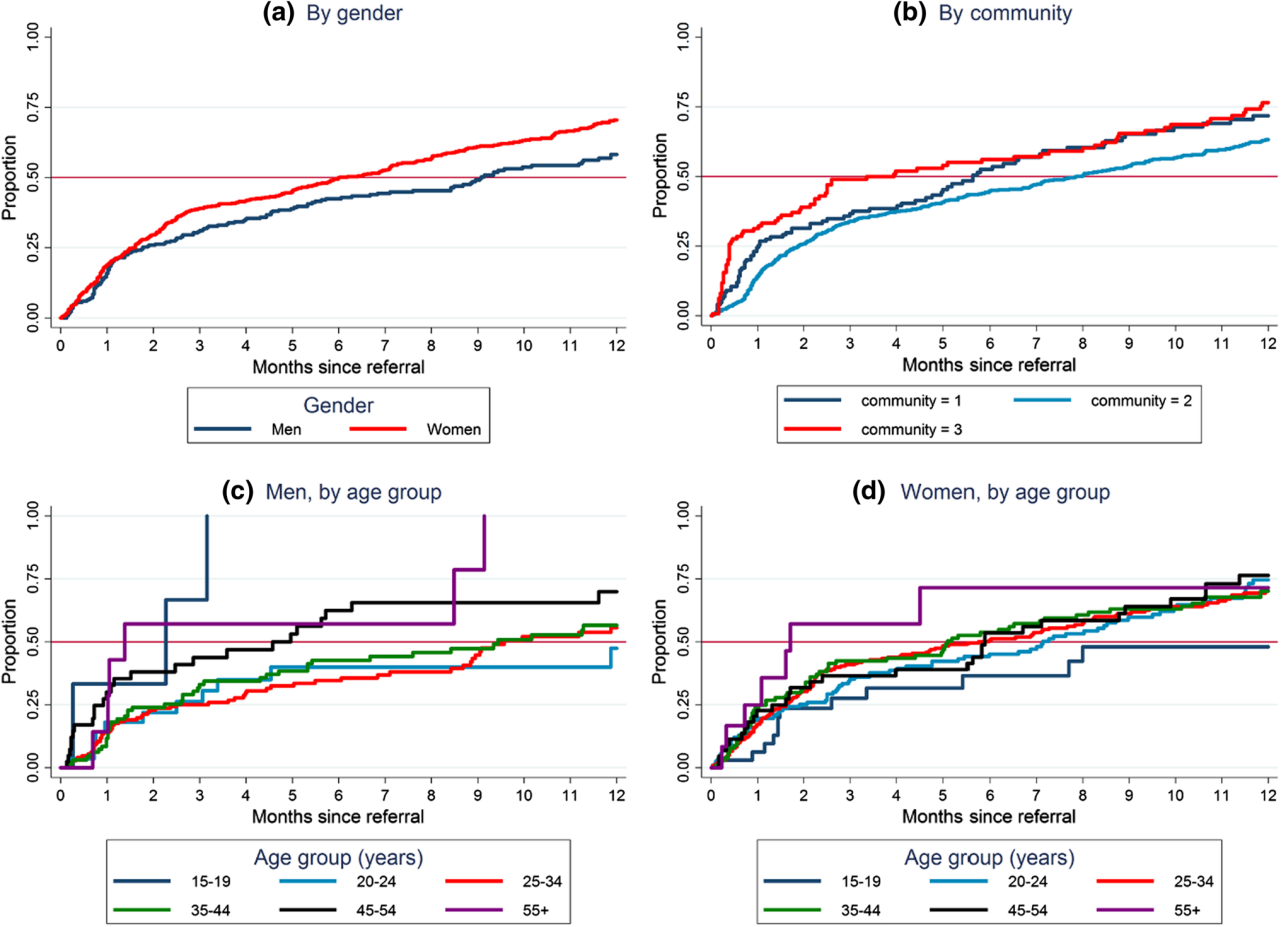
Time from CHiP referral to ART initiation in South Africa in Round 2, by gender, community, and age group

For South Africa, findings are summarised in Table 2 and Fig. 3. The median time to ART initiation was shorter for women than men (6.0 and 9.1 months respectively, p = 0.006). There was strong evidence of differences among communities (p = 0.007) with the median time to ART initiation being 5.6, 7.9, and 3.9 in communities 1, 2 and 3 respectively. Among men, it was shorter for older men aged ≥ 45 years compared with those aged 25–29 years, and longer for women aged 15–19 years compared to those aged 25–29 years.

### Time from Linkage to Care (attendance at clinic) to ART Initiation—Patterns by Gender, Community, and Age Group

The estimated time from linkage to care to ART initiation was similar for men and women, and across communities, in Zambia. In South Africa there was evidence that this time was shorter in communities 1 and 3 compared with community 2, but the difference was relatively small (shown in Supplementary Table 2). In both countries, for both men and women there was no evidence of differences by age group (data not shown).

### Qualitative Findings

Our findings show that there are differences in time to ART initiation between communities and these can be categorised by: distance between household and clinic; levels of socio-economic status and employment; reported clinic staff workload at the health care facility; and the fear of being seen accessing services affecting time to ART initiation.

For example, the health facility in Community 1, is located quite far from the town centre, the population the facility serves is relatively educated, middle-class with some PLHIV working within the area. This one ART clinic is not particularly congested (according to users), because of a lower overall population density compared to other communities. In contrast, in Community 4, the health care facility is located close to the town centre. The population accessing care there are mostly of lower socio-economic class, engaged in the informal economy and often involved in mobile livelihoods (for example: cross country-border trade and fishing). Women traders in this community work long days in markets or selling by the roadside and are involved in trade which also involves periods away from the community, restricting their opportunities to attend the clinic. Alcohol use is reported to be high in this community. The clinic is congested (waiting time was usually > 3 h).

Similarly, in South Africa there is an observed association between clinic burden (those serving over 1000 clients a month are considered over-burdened), and a slower time to ART initiation. Community 3, where there is a shorter average time to start ART, is a relatively small community with lower HIV prevalence and fewer people initiating ART each month. In contrast, community 2 is a relatively large ‘gate-way’ community for people entering Cape Town from Eastern Cape searching for employment opportunities and has a relatively high HIV prevalence, and the clinic is busy. Even before the HPTN 071 (PopART) trial began the clinic had a much larger number of clients on ART than the other two communities. Thus, it is notable that in both countries, the populations slowest to initiate ART are those with transient populations (engaged in mobile livelihoods) and congested health facilities.

Health system, structural and social reasons for taking time to link to care are summarised in Table 3 (below). We have grouped the summary findings in the table by health care facility, community, household and individual factors, although several reasons listed are cross-cutting. We now elaborate on some of the reasons given for delaying treatment listed in Table 3, including the gender and age factors apparent in the process data, using *italics* to highlight reasons mentioned in the table, to ease cross-referencing.

**Table 3:** Reasons not to start ART quickly in seven communities (four Zambia, three South Africa)

Reasons	
Health services factors	*Congestion* Long waiting times (> 3 h) affecting livelihood demands Clinic staff perceived to open clinic on time but start work late Frustration with having to be at the clinic early in the morning to avoid long queues Nursing staff perceived to take extended tea breaks and lunch (South Africa)^a^
	*The risk of ‘being seen’* PLWH anticipated being seen by others who knew them at the clinic Fear of involuntary disclosure when accessing services in designated areas of HIV care HIV services in facilities also considered to offer privacy *once* in designated areas
	*Quality of HIV testing/ART* Some rumours about false positives Some lack of trust in the accuracy of HIV test results Some (limited) rumours about variation in the quality of the ART (in areas with predominantly black community members thought to receive ‘weaker’ ART) (South Africa) Some rumours of clinic staff perceived to be reimbursed for each HIV-positive diagnosis (South Africa)
	*Record-keeping* Lost clinic folders/CD4 results at facilities Locating files increased waiting time Frustration with misplaced files making PLWH leave before collecting the drugs
	*HIV service delivery structures/processes* ART initiation procedures lengthy (re-testing for HIV & counselling) and unclear (Zambia) Clinic operating hours limited (ART services provided in the week day morning) (Zambia) Lack of laboratory reagents/apparatus for necessary tests prior to ART initiation delays start of treatment. (Zambia) Requirement to provide identification or proof or residence problematic for people living in informal housing structures with no formal physical addresses (South Africa)
	*Anticipated difficulties of re-engaging with HIV services* Anticipation of being admonished by health care workers for missing treatment/defaulting Worry about adhering to appointment dates alongside competing alternative responsibilities (livelihood, family responsibility)
	*Informal charges* Informal costs to access care: missing work/loss of income, travel cost to clinic Offering informal incentives (money) to both professional and lay health care workers a strategy of getting around congestion/avoiding queuing (Zambia)
	*Health worker attitudes* Some negative remarks made by health care workers when providing services Some unsatisfactory health care worker and client relationships Preferential treatment to some clients
Community Factors	*Gender* More women accessing HIV services than men (linked to antenatal care access) Men considered busy and difficult to link to care
	*Community Health Worker relationships* Community health workers resident in the community, so some fears of confidentiality breaches Some tensions between lay workers and clinic staff (South Africa)
	*Alternative treatment options* Faith healing—belief that HIV can be cured through prayer (Zambia) Herbal remedies and immune boosters used to improve health Traditional medicine is taken as supportive care (South Africa) Some participants use drugs (‘tik’ [crystal meth], marijuana) to self-medicate or as a form of escapism, which can delay treatment access (South Africa)
	*Other priorities/concerns/illnesses* Competing concerns such as: securing income, substance abuse, drug use Other health conditions (e.g. TB, diabetes) Crime (often dangerous/violent) poses challenges (South Africa) Some reports of theft of anti-retroviral drugs for recreational drug use (South Africa) Isolation due to fear of disclosure to other community members
Household Factors	*Food* Food insecurity affecting uptake of ART (perception that when taking a strong drug like ART one needs to take it with food and stick to a special diet) Household survival factors contribute delays in accessing treatment (food availability depends on tenuous and variable income)
	*Livelihood* Employment scarce and some people hesitant to miss work in order to attend the clinic Labour migration potentially impacts the decision to link to care
	*Age* Adolescents needing guardian’s consent to start ART Lay workers delaying disclosure of HIV status to adolescents in the absence of guardians (Zambia)
	*Partner support/couple and gender dynamics* Difference in opinions among couples on whether to start ART or not Anticipated negative reaction to women’s treatment initiation (threats of divorce, violence, abuse, lack of support, disclosure to wider community) can contribute to delays in treatment access Women’s dependence on husbands affecting own decision-making capacity especially in Zambia
Individual Factors	*Waiting to be sick/feeling fine/managing without* Current state of ‘feeling well’ seen as motivation not to take medication yet Conflict with earlier messaging from health facilities that one needed to ‘be sick’ (have low CD 4 levels) to be able to access treatment Taking care of self through appropriate diet and use of herbal remedies without feeling sick gives indication of managing without ART Influence from other PLWH who have not taken ART despite living with HIV for many years Some perceptions that medical staff will not be serious about treatment if clients do not present as ‘sick’ (South Africa)
	*Acceptance/readiness/denial* Non-acceptance of HIV status affects linkage to care Needing more time to accept HIV status after HIV diagnosis Not being ready to commit to lifelong treatment Some health care workers felt it was right to give time to PLWH to accept their status and be ready to take up treatment
	*Gender* Intimate partner violence: some women fear accessing treatment because of partner’s attitude and behaviour Men and women of different ages respond to offers of testing and treatment differently
	*Marginal identity* Transient people (fisherfolk, traders, seasonal farm workers) face challenges in accessing care Sex workers, MSM, transgender individuals express fear of anticipated stigma at facilities (from community and some health staff) (South Africa)
	*Substance use* Alcohol use/abuse affects decisions to link to care and adhere to treatment Alcohol seen as competing with ART treatment regimens—‘choose’ between substances and treatment because belief cannot use substances and be on treatment
	*Impact of ART on body & for life* Fears of implications for life-long commitment to medication Pill burden seen as potentially overwhelming (often based on knowledge of number of pills taken by PLHIV in the past) Some PLWH expressed needing to take treatment ‘breaks’ but discouraged to do so by facility staff

^a^Where a reason was given in one country, this is marked with the country name

### Health Service Factors

The *fear of being seen at a clinic* accessing services, including starting and collecting ART, was a deterrent mentioned by participants in both countries in all seven communities. The stigma associated with accessing HIV-treatment services included the anticipated stigma of people in the community finding out about clinic attendance. For example, one couple in South Africa complained that being familiar with health staff at the local clinic stopped them from initiating treatment because they feared the resident health staff would disclose their status to others. Others were concerned about being seen by neighbours and friends waiting in parts of the clinic associated with HIV care.

Other factors given for not linking to care included confusion over changing and seemingly-conflicting messages from health workers about when to start ART because of shifting guidelines. Others had been advised by health workers to eat nutritious food with their treatment which they could not afford: a 47-year old woman LHIV in Zambia recalled how health workers told her to buy fruits, but she and her husband could not always pay rent for the room they lived in, so could not spend more money on food. As a result, they had not linked to care.

There were worries about the stigma of being seen once on treatment. A Zambian older man (Z10) recounted, “some people fear queuing at the clinic to collect drugs”. He attributed *congestion* and lack of privacy in health facilities as contributing to making accessing drugs ‘public’, and remarked, “Accessing drugs should be private or the government should find another way of providing HIV drugs”. In Zambia, respondents accessing care in the busy health facilities spoke about *long waiting times* (over 3 h), with staff and clients sometimes being rude to each other. A few respondents expressed concern that there was preferential treatment for some clients and some who used their authority and status to go ahead in the queue.

One woman in South Africa (S16) talked about the discomfort she felt about her nearby health care facility, a factor which contributed to other reasons she gave for not starting ART.

The clinic staff like to gossip, I mean, I don’t feel comfortable with them…. I would probably take ART somewhere else, these people from here are not right…. They know me and they like gossiping outside.

*Health worker attitudes* were mentioned by others with some fearing being reprimanded by health care workers for failing to take treatment. Stories of people who had been scolded at the health care facility for not adhering to treatment when they tried to access care after a gap in treatment, were recounted by people in both countries. These were all factors which made access difficult once someone had linked to care, and deterred others who were yet to start ART.

While health system factors deterred some PLHIV from accessing treatment, family and community views also influenced uptake, often in association with the stigmatising attitudes in the community about those accessing HIV-care services.

### Community and Household Factors

In both countries people were often juggling their *livelihood needs* with starting ART, fearing time lost accessing care when they needed to work [45]. Some PLHIV who were the main breadwinners and had not started ART, would rather use their strength to earn an income and continue providing for their family, while they were feeling healthy, than go for treatment. Sometimes making a living involved long days in the market or months away in fishing camps, farming, mining or the road and construction camps.

A woman sex worker living with HIV and not yet on ART in South Africa (S14) commented that she had to use her time to look for clients, and that having no clients meant she earned no money and there would therefore be no food at home. When she was pregnant she used antiretroviral drugs for the prevention of mother to child transmission of HIV (PMTCT), because her partner used to support her then, so that she did not infect the unborn baby. However, she did not continue with ART after PMTCT. She said she has not been sick ever since she found out about her status so did not see the need for treatment.

Poverty and *lack of food* influenced the decision of some people to delay treatment uptake, as mentioned above. A 41-year-old woman in South Africa (S14) commented:

So, you have to take treatment every day. So, can you take treatment on an empty stomach? Really? Because even if you are working, there is this maybe two weeks before payday you don’t have anything to eat. So, you’ll have to take a glass of water and pills and go to bed without anything you see. That’s why HIV people, most of the cases, they just default and go drinking [alcohol].

Maturity and *age* engendered responsibility and autonomy in both women and men, sometimes, but not always, making it easier for older people to decide to start ART. The responsibility of being a provider prompted one 48-year-old Zambian man (Z10) to take treatment; “if I take treatment, I will be able to look after my family. If I do not take the treatment it is the family that will suffer”.

Disclosure of HIV status to *partners*, between women to their male spouses (in Zambia) was cited as a reason to delay attending care and starting ART. One strategy was to hold off disclosing an HIV-positive status to a partner until they also tested for HIV. In Zambia, women were more likely to be married and living with their husbands than in South Africa. Sometimes, it was up to men in the household to decide if women should start ART or not. Many Zambian women participants spoke of experiencing *gender-based violence* (physical and verbal), because of the stigma of HIV-infection and assumptions on the part of the male partner about the woman’s sexual behaviour or the woman’s accusations against the man and his behaviour that could have resulted in her infection. Thus, women in Zambia appeared constrained by male hegemony. This was reflected by a younger woman on ART (Z2) who talked about women in her community being beaten and insulted by their husbands for questioning the man’s behaviour, and then hiding indoors for fear of people seeing their physical injuries. Another woman who was HIV-negative (Z7) relayed how violence and the threat of violence can affect a woman’s decision to take treatment:

a man beat up his wife because she started ART, although even he tested HIV-positive. He is one of the people who says ‘HIV and its treatment is a lie, they just cheat us about these drugs’. So right now the woman is sick, she is bedridden.

Although fewer women in our study population were married in South Africa than in Zambia, amongst those who were, participants said it was harder for a woman to start treatment if her husband had not begun ART. A 21-year-old women living with HIV whose husband was in prison said:

I disclosed my status to my in-laws, they advised that I wait for my husband to come out of jail before I could start ART. I will wait for him to be released from prison to get permission to start ART. If he refuses, then I won’t start.

In both countries, *gender* was an important factor in treatment access. Women had more frequent contact with the health facility than men through pregnancy and child health needs, and the health facility was seen more as women’s space. However, whereas young men’s freedom to move around the community and engage in informal livelihood activities sanctioned and facilitated their use of the clinic in Zambia, in South Africa, men were more likely to resist going to the clinic. A South African man living with HIV (S21) was initiated onto ART but quickly stopped taking it. He was a gang member and an active drug user (taking Mandrax [methaqualone] and Tik [crystal meth]), he had recently been in prison and continued to be involved in criminal activities. As in this example, past-history, gang culture, alcohol and drugs were said by participants to mean that men did not prioritise their health, as also shown in findings from other research [34, 46, 47].

### Individual Factors

Despite community engagement and messaging on the benefits of immediate ART, many people had internalised previous health messaging about treatment being appropriate only for those who were sick, and were cautious about accepting the change in advice [38]. Therefore, some considered *immediate treatment appropriate for those who are ill*, pregnant or in a discordant relationship. A Zambian 51-year-old man (Z7) explained: “

When I tested HIV-positive I did not see it fit to start ART because I was not feeling sick, I did not have any pain and I did not feel weak because I used to even lift heavy weights. So, I felt that even if I start taking treatment, it will not change anything.

For other PLHIV, although understanding the health benefits of antiretroviral treatment and expressing gratefulness for wider ART access, people talked of the time needed to ‘*accept’ the diagnosis*, to contemplate disclosure, household living and clinic options, and to consider the *impact of treatment on the body*, livelihood and relationships. People often talked about the need to `process’ the information that they were HIV-positive. A 21-year-old man (S13) who tested HIV-positive with one of the CHiPs teams in April 2017, said that when he heard he was HIV-positive “I couldn’t believe it”. He was previously HIV-negative in October 2016 and his disbelief prevented him from accessing care. He also said he was kept busy with other things which kept him from the clinic.

While some respondents talked about the fear of starting ART, they did not always indicate concern that ART would itself have an adverse impact on their bodies. Instead, concern often focused on the worries of being forced to interrupt ART due to drug stock-outs, a situation beyond their control.

Denial, anger, sadness, and questioning were common responses amongst adolescents living with HIV when they learnt of their status. One 21-year-old perinatally-infected man (Z2), who had stopped taking ART, said that he ‘did not believe in ART’. A 22-year-old woman living with HIV (Z8) shared her experience when she first found out she was HIV-positive:

I felt bad, I cried I won’t lie. I cried because I wasn’t expecting [it], I had a lot of thoughts that came that I should kill myself and do other things, […] then I thought about home where I stay, how they were going to receive me looking at how I am living…

For younger people, it was said to be harder to accept your status. A woman in South Africa (S18) mentioned that young people feel they are *too young to be taking treatment for the rest of their life* and they do not have the discipline to be taking ART every day. Another Zambian 17-year-old girl living with HIV (Z7) said when she first found out she had HIV:

I felt very bad, whereby many questions came to my head like, was I born to be an ARV taker or maybe to be addicted because my life relies on these same ARVs. It was very hard for me.

### Reasons to Start Treatment Promptly

Yet, despite the many reasons given for delaying starting care, participants in both countries did recognise the importance of starting treatment straightaway, at least for some people. These reasons, summarised in Table 4, point to the value placed on health care facility access and the importance of the support of family and friends as well as being well enough to make a living to contribute to the family.

**Table 4:** Reasons to start ART promptly in seven communities (four Zambia, three South Africa)

Reasons to start ART	
Health services factors	Positive perception and previous experience of health facility and services seen as facilitating care Previous (affirmative) relationships with staff at facilities Home testing, information, linkage to care and support from CHWs (including CHiPs) Availability of adherence clubs Preferential treatment due to their occupation or status (police, school students)
Key health motivations	Illness: feeling sick, prolonged severe ill health Co-infection, i.e. HIV & TB impacting on health Previous HIV-related death in the household/family Pregnancy (PMTCT) ART viewed as way to maintain or regain physical health Family responsibility: being well to take care of family Keeping partner safe/negative (treatment as prevention)
Familial and social factors	Relatives/spouses (including HIV + partners and family) facilitating treatment initiation Families providing support: transport, social support, adherence support Social networks as support structures: church, friends, loan groups Friends collecting treatment on behalf of PLWH

Past experiences did play a part in the decision to start treatment for some, even if they were not yet ill themselves. Seeing a family member grow weak and die because of HIV, and in some cases remembering episodes of ill health as a child, prompted access to treatment. A 21-year-old woman (Z2) commented:

I have experienced this, not myself but with my late mum when she defaulted, she then stopped taking her medication and this cost her so much such that she had meningitis and her kidney could not function, so I learned from her. So, I started, and I can’t stop because what happened to her can also happen to me.

## Discussion

Over the first 2 years of delivering the HPTN 071 (PopART) intervention we observed steep reductions in the time taken from community household HIV testing to linking to care and starting ART. However, ~ 30% of individuals had not initiated ART by 12 months after testing.

Whilst immediate ART for all PLHIV and “same day ART start” have been shown to enhance health outcomes [48] when delivered at the clinic, and is being advocated as a clinic-based approach to facilitate ART-start in HIV programmes [49], home-based and community wide programmes offering HIV testing require additional time to link to care and treatment in a clinic [50]. A meta-analysis has identified that the best way to reach coverage approaching the first UNAIDS 90% target is through community models of testing [51], an observation supported by more recent studies [15, 36, 52]. This approach, as well as HIV-self-testing [53, 54], means there will still be a gap between the initial test result, confirmation of HIV-positive status and ART initiation. Therefore, the current programmatic emphasis supported by policy makers and funders [55] on “same day test and start”, whilst potentially expediting the time required to initiate ART for those testing HIV-positive, may not be applicable to those testing within the community, unless community-based ART initiation is implemented [50].

The delay or loss of PLHIV to starting ART is a critical challenge to delivering a successful HIV treatment and prevention cascade. The reasons for this are multi-factorial, as we describe above and highlighted in a previously reported nested case control study in HPTN 071 (PopART) [56]. Community engagement, mobilisation and messaging were an integral part of the HPTN 071 (PopART) trial and were refined over time (as described above) to encourage timely linkage to care. The CHiPs workers became more accepted and familiar within the communities and the health care facilities similarly became more comfortable offering universal ART, as we have described elsewhere [56]. Despite these enhancements, there were key groups of PLHIV that chose not to link to care or start ART, in keeping with findings from other similar trials [12]. We found that in South Africa, men tended to link to care and initiate ART more slowly than women, as has been noted by other investigators [46, 47]). Yet, our data from Zambia which show that men, particularly older men, linked to care more quickly than women, runs counter to the large body of work highlighting men lagging behind in accessing care [57]. We observed that one reason for this difference in Zambia was because some women who linked to care faced negative repercussions from their partners, particularly younger and middle-aged women. It may also be that a successful campaign to encourage men to test and link to care in the context of also accessing care for other conditions, which was provided as part of the PopART community engagement activities in Zambia, increased uptake [42].

The fear of ‘being seen’ accessing treatment remains a threat, particularly for marginal and higher status groups. The stigmatising attitudes in communities are enacted through the concerns expressed by PLHIV about engagement with the health system, resulting in fearing being seen and possible disclosure of their HIV-status. This risk of being identified, and associated stigma, was exacerbated by the way the clinics were usually organised (with separate areas for PLHIV to wait) which we describe elsewhere [40], and (in most but not all clinics) high numbers of clients, over-crowding, long waiting times and congestion [ibid]. These health system factors interact with stigma about HIV, increasing perceived barriers to care for some PLHIV. The concerns around stigma are a reflection of wider community level stigma about HIV in these communities [35]. In addition, experience with overstretched health services and busy workers means that PLHIV are aware that a commitment to life-long treatment involves routine and lengthy interactions with health care services and raises questions and fears for the sustainability of treatment.

Despite the encouraging shortening of time to ART initiation across the two Rounds reported in this paper there is still a substantial number who do not link to care within the first 12 months; there are differences between communities as well as household and individual reasons for delaying treatment, as we describe above. The significant differences across communities were related to degrees of mobility, presence of middle-class residents, employment options, and congestion at the government health facility. This corresponds with earlier mixed method analysis of first year intervention uptake in the four Zambian Arm A intervention communities [56] and underscores the influence of the local context on the ability of PLHIV to link to care. Our analysis identified some groups (for example, younger men and mobile populations) as often being slower to start ART, probably because the health service delivery mechanisms are not appropriate to their needs. These observations are in keeping with the findings from other studies [12, 16, 22, 28, 58]. As has been demonstrated recently with the findings for the ANRS 12249 TasP Cluster-Randomised Trial, even in the controlled environment of a trial, ensuring prompt treatment may be challenging [15].

Given health system constraints, as well as PLHIV decision-making, circumstances and stigma, barriers will continue to affect uptake. These barriers may be addressed to some extent by the current motivation for differentiated models of care, which aims to adapt health service provision to client needs through less frequent clinic attendance for people who are stable on ART, and providing non-facility based outlets for ART delivery closer to people’s homes [59, 60].

There is a recognised HIV continuum of care or “cascade” that requires at least three critical steps to ensure delivery of successful ART to all PLHIV. In the original care cascade [61, 62], Stage 2 was retention in pre-ART care until the PLHIV became eligible for ART. Fox and Rosen [63] argue that Stage 2, is now redundant because there should be no delay between diagnosis (Stage 1) and eligibility to start ART (Stage 3). Yet, as our findings show there remain significant gaps in the HIV care continuum, challenging ART coverage. While the reduction in Stage 2 is logical for those testing within a health care facility, a brief `pre-ART’ period may serve as an opportunity to come to terms with HIV status prior to commencing ART [64]. This time, in particular for asymptomatic PLHIV, may serve an important role. Indeed, in the longer-term, immediate same day ART may need to be complemented with other options, to ensure the sustainability of the approach. While shortening the gap between testing and treatment is clinically important, it is an imperative that support for people for their treatment decision-making, and facilitating access to care, is provided.

Our findings show that PLHIV often delayed starting ART because of issues to do with the quality of care available and the stigma associated with accessing care. If the quality of and access to care were to improve (including re-arranging service access to integrate HIV with other care) and stigma were to reduce, fewer people may delay starting treatment. That said, there may remain individuals who require some time to come to terms with the challenges of living with HIV prior to starting therapy, a finding corroborated in other settings [34, 65–68], although in the PopART study, because of the home-based testing model, same day ART start was not an option. Despite the multiple operational challenges to deliver optimal treatment and care for all PLHIV in resource limited high burden settings, more work is needed on changing messaging, stigmatising attitudes towards PLHIV and capacity at health care facilities. Respect for individual choice means that a differentiated model of care [69, 70], with multiple options, might be the best way to deliver the breadth of ART coverage necessary to retain all PLHIV in care and get to zero new infections and zero deaths from HIV-related illness.

## Conclusion

To enhance ART coverage amongst all PLHIV, timely linkage to care is necessary. However, for programmes offering community-based HIV-testing, unless community ART initiation is offered, there is an inevitable lag between HIV testing and ART initiation. Our findings suggest that for some PLHIV a period of adjustment maybe important for the individual newly-diagnosed and sometimes also for their family. We need to work on interventions that ensure clear messaging about the benefits or ART, that limit stigma, structural barriers, overcrowding and long waiting times, where they exist. And we need to promote prompt treatment whilst allowing people, if necessary, to take time to adjust and think, without compromising their health outcomes. The time and support needed may be different for women and men, in different local contexts and for people of different ages.

## Electronic supplementary material

Below is the link to the electronic supplementary material.

Supplementary material 1 (DOCX 23 kb)
