# Supplementary material for: Understanding the Time Needed to Link to Care and Start ART in Seven HPTN 071 (PopART) Study Communities in Zambia and South Africa
Source: AIDS Behav. 2018 Nov 10;23(4):929–46. doi: 10.1007/s10461-018-2335-7 (PMC6458981; doi:10.1007/s10461-018-2335-7)
Supplement: Supplementary file 1 — Supplementary material 1 (DOCX 23 kb) [file 10461_2018_2335_MOESM1_ESM.docx]

**Supp. Table 1 - Time to link to HIV care after first CHiP referral to HIV care in Round 2 – Zambia and South Africa**

|  | **Number referred to HIV care** | **Linked to HIV care (%)^1^** | | | | **Hazard ratio, unadjusted** | **Hazard ratio, adjusted^2^** | **95% CI** |
| --- | --- | --- | --- | --- | --- | --- | --- | --- |
| **Zambia** |  | **1 month** | **3 months** | **6 months** | **12 months** |  |  |  |
| **Overall** | 3,435 | 30  95%CI 28-32  *(n=2,585)^3^* | 45  95%CI 43-47  *(n=2,388)* | **57**  95%CI 55-59  *(n=2,242)* | **71**  95%CI 69-73  *(n=2,042)* |  |  |  |
| **Gender** |  |  |  |  |  |  |  |  |
| Men | 1,117 | 26 | 44 | **56** | **71** | 1 (ref) | **1 (ref)** | *P=0.002^4^* |
| Women | 2,318 | 21 | 37 | **48** | **65** | 0.85 | **0.84** | **0.76-0.94** |
| **Community** |  |  |  |  |  |  |  |  |
| 1 | 256 | 46 | 61 | **71** | **78** | 1.47 | **1.49** | **1.25-1.78** |
| 2 | 856 | 30 | 46 | **58** | **71** | 0.98 | **0.97** | **0.86-1.09** |
| 3 | 1,775 | 31 | 46 | **57** | **72** | 1 (ref) | **1 (ref)** | *P<0.001* |
| 4 | 548 | 19 | 33 | **45** | **62** | 0.71 | **0.71** | **0.61-0.83** |
|  |  |  |  |  |  |  |  |  |
| **South Africa** |  |  |  |  |  |  |  |  |
| **Overall** | 1,262 | 31  95%CI 28-34  *(n=875)^3^* | 48  95%CI 45-52  *(n=830)* | **60**  95%CI 57-64  *(n=782)* | **79**  95%CI 76-82  *(n=732)* |  |  |  |
| **Gender** |  |  |  |  |  |  |  |  |
| Men | 436 | 30 | 45 | **55** | **73** | 1 (ref) | **1 (ref)** | *P=0.03* |
| Women | 826 | 31 | 50 | **63** | **82** | 1.24 | **1.22** | **1.02-1.45** |
| **Community** |  |  |  |  |  |  |  |  |
| 1 | 204 | 33 | 46 | **64** | **82** | 1.14 | **1.08** | **0.87-1.35** |
| 2 | 909 | 29 | 47 | **59** | **77** | 1 (ref) | **1 (ref)** | *P=0.21* |
| 3 | 149 | 37 | 57 | **65** | **86** | 1.27 | **1.23** | **0.98-1.55** |

1 Estimated from “time to event” analysis; 2 For overall comparison of women with men, adjusted hazard ratios are obtained from a multivariable Cox regression model including community, age group, and gender; for overall comparison among communities, adjusted hazard ratios are obtained from a multivariable Cox regression model including community, gender, and gender-specific hazard ratios for age group; 3 Number who either linked to HIV care within 1 month after referral or have a follow-up visit ≥1 month after referral, and similarly for other time points (3, 6, 12 months after referral); 4 P-values are from Cox regression, from likelihood ratio tests of whether there is evidence of association between an individual characteristic (e.g. gender, or the community in which an individual lives) and the outcome of “time to link to HIV care”.

**Supp. Table 2 - Time from linkage to HIV care to ART initiation in Round 2 – Zambia and South Africa**

|  | **Number linked to HIV care** | **ART initiated (%)^1^** | | | | **Hazard ratio, unadjusted** | **Hazard ratio, adjusted^2^** | **95% CI** |
| --- | --- | --- | --- | --- | --- | --- | --- | --- |
| **Zambia** |  | **1 month** | **3 months** | **6 months** | **12 months** |  |  |  |
| **Overall** | 1,774 | **78**  95%CI 76-80  *(n=1,736)^3^* | **90**  95%CI 88-91  *(n=1,712)* | **94**  95%CI 93-95  *(n=1,700)* | **98**  95%CI 98-99  *(n=1,686)* |  |  |  |
| **Gender** |  |  |  |  |  |  |  |  |
| Men | 558 | **80** | **92** | 96 | 99 | 1 (ref) | **1 (ref)** | *P=0.25^4^* |
| Women | 1,216 | **77** | **89** | 93 | 98 | 0.94 | **0.94** | **0.84-1.05** |
| **Community** |  |  |  |  |  |  |  |  |
| 1 | 144 | **82** | **93** | 96 | 99 | 1.19 | **1.20** | **1.00-1.44** |
| 2 | 439 | **78** | **90** | 94 | 99 | 1.02 | **1.02** | **0.91-1.15** |
| 3 | 962 | **79** | **90** | 95 | 99 | 1 (ref) | **1 (ref)** | *P=0.03* |
| 4 | 229 | **70** | **86** | 92 | 96 | 0.86 | **0.86** | **0.74-1.00** |
|  |  |  |  |  |  |  |  |  |
| **South Africa** |  |  |  |  |  |  |  |  |
| **Overall** | 677 | **64**  95%CI 60-68  *(n=614)^3^* | **81**  95%CI 78-84  *(n=599)* | **87**  95%CI 84-89  *(n=590)* | **91**  95%CI 89-94  *(n=577)* |  |  |  |
| **Gender** |  |  |  |  |  |  |  |  |
| Men | 221 | **63** | **80** | 84 | 91 | 1 (ref) | **1 (ref)** | *P=0.14* |
| Women | 456 | **64** | **82** | 88 | 92 | 1.09 | **1.15** | **0.95-1.39** |
| **Community** |  |  |  |  |  |  |  |  |
| 1 | 110 | **74** | **90** | 91 | 96 | 1.33 | **1.28** | **1.02-1.61** |
| 2 | 469 | **59** | **79** | 85 | 90 | 1 (ref) | **1 (ref)** | *P=0.005* |
| 3 | 98 | **76** | **82** | 90 | 95 | 1.43 | **1.43** | **1.13-1.82** |

1 Estimated from “time to event” analysis; 2 For overall comparison of women with men, adjusted hazard ratios are obtained from a multivariable Cox regression model including community, age group, and gender; for overall comparison among communities, adjusted hazard ratios are obtained from a multivariable Cox regression model including community, gender, and gender-specific hazard ratios for age group; 3 Number who either started ART within 1 month after referral or have a follow-up visit ≥1 month after the estimated date of linkage to HIV care, and similarly for other time points (3, 6, 12 months after referral); 4 P-values are from Cox regression, from likelihood ratio tests of whether there is evidence of association between an individual characteristic (e.g. gender, or the community in which an individual lives) and the outcome of “time from linkage to HIV care to ART initiation”.
